# Psychiatric symptoms of wife of military personel who died in combat: The role of meaning in life and attachment styles

**DOI:** 10.1192/j.eurpsy.2021.2036

**Published:** 2021-08-13

**Authors:** H. Korkmaz, B. Güloğlu

**Affiliations:** 1 Psychological Counseling And Guidance, Bahçeşehir University, Beşiktaş/İstanbul, Turkey; 2 Psychological Counseling, Bahcesehir University, Istanbul, Turkey

**Keywords:** attachment styles, The Meaning in Life, Wife of Military Personel, Psychiatric symptoms

## Abstract

**Introduction:**

Traumatic experiences has a key role on the mental health of individuals. Turkish Armed Forces has been involved in various combat in and out of the country over the years. Individuals who are able to find meaning after a negative life events are better overcome their issues and return to their positive functioning. Unhealthy attachment styles has been observed more in clinical samples than healthy attachment styles.

**Objectives:**

The aim of this study was to investigate the predictive role of attachment styles and meaning in life on psychiatric symptoms among wife of military personel who lost their lifes.

**Methods:**

74 women who lost their husband in combat to the study. Their age was between 21 and 74, with the mean of 46.93. 60 (75.9%) participants had a child when they lost their husband. 63 (79.7%) of them hasn’t been married again. Brief Symptom Inventory, Meaning in Life Scale and Relationship Scales Questtionnare were used to collect the data. Five different regression analysis was conducted.

**Results:**

Finding meaning in life, dismissing and preoccupied attachment sytles predicted depression (R^2^= 51.8%). Finding meaning in life and fearful attachment styles predicted anxiety (R^2^= 46.2%). Finding meaning in life and fearful attachment styles predicted negative identity (R^2^= 51.1%). Finding meaning in life and dismissing attachment styles predicted hostility (R^2^= 50.4%) and somatization (R^2^= 57%).

**Conclusions:**

Meaning in life has a protective role in the development of any psychopathologies whereas insecure attachment styles are risk factor.

**Disclosure:**

No significant relationships.

